# (*E*)-4-Chloro-2-{[4-(di­methyl­amino)­benzyl­idene]amino}­phenol

**DOI:** 10.1107/S1600536814008873

**Published:** 2014-04-26

**Authors:** Nadir Ghichi, Mohamed Amine Benaouida, Ali Benosmane, Ali Benboudiaf, Hocine Merazig

**Affiliations:** a Unité de Recherche de Chimie de l’Environnement et Moléculaire Structurale, (CHEMS), Faculté des Sciences Exactes, Département de Chimie, Université Constantine 1, Algeria

## Abstract

In the title aromatic Schiff base compound, C_15_H_15_ClN_2_O, the mol­ecule exists in a *trans* conformation with respect to the C=N bond. The dihedral angle between the benzene rings is 14.49 (6)°. In the crystal, weak C—H⋯π inter­actions link mol­ecules into supra­molecular chains propagated along the *a*-axis direction.

## Related literature   

For the use of Schiff bases in synthesis, see: Arora *et al.* (2002 ▶). For their use as biological, analytical, polymer and liquid crystalline materials, see: Tanaka & Shiraishi (2000 ▶). Schiff bases have been reported to show anti­bacterial (Jarrahpour & Khalili, 2006 ▶; Jarrahpour *et al.*, 2004 ▶; El-masry *et al.*, 2000 ▶), anti­fungal (More *et al.*, 2001 ▶; Singh & Dash, 1988 ▶), anti­cancer (Desai *et al.*, 2001 ▶; Phatak *et al.*, 2000 ▶) and herbicidal activity (Samadhiya & Halve, 2001 ▶). For related structures, see: Akkurt *et al.* (2005 ▶, 2008 ▶).
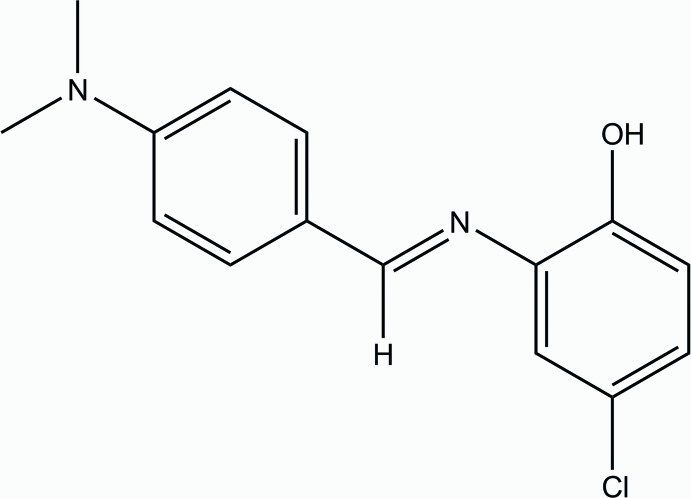


## Experimental   

### 

#### Crystal data  

C_15_H_15_ClN_2_O
*M* *_r_* = 274.74
Orthorhombic, 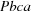

*a* = 7.411 (5) Å
*b* = 12.314 (5) Å
*c* = 29.684 (5) Å
*V* = 2709 (2) Å^3^
*Z* = 8
Mo *K*α radiation
μ = 0.28 mm^−1^
*T* = 293 K
0.03 × 0.02 × 0.01 mm

#### Data collection  

Bruker APEXII CCD diffractometer
14319 measured reflections
2346 independent reflections
1895 reflections with *I* > 2σ(*I*)
*R*_int_ = 0.028

#### Refinement  

*R*[*F* ^2^ > 2σ(*F* ^2^)] = 0.043
*wR*(*F* ^2^) = 0.102
*S* = 1.10
2346 reflections
172 parameters
H-atom parameters constrained
Δρ_max_ = 0.20 e Å^−3^
Δρ_min_ = −0.20 e Å^−3^

### 

Data collection: *APEX2* (Bruker, 2007 ▶); cell refinement: *SAINT* (Bruker, 2007 ▶); data reduction: *SAINT*; program(s) used to solve structure: *SHELXS97* (Sheldrick, 2008 ▶); program(s) used to refine structure: *SHELXL97* (Sheldrick, 2008 ▶); molecular graphics: *ORTEP-3 for Windows* (Farrugia, 2012 ▶); software used to prepare material for publication: *WinGX* (Farrugia, 2012 ▶).

## Supplementary Material

Crystal structure: contains datablock(s) global, I. DOI: 10.1107/S1600536814008873/xu5785sup1.cif

Structure factors: contains datablock(s) I. DOI: 10.1107/S1600536814008873/xu5785Isup2.hkl

Supporting information file. DOI: 10.1107/S1600536814008873/xu5785Isup3.cml

Additional supporting information:  crystallographic information; 3D view; checkCIF report

## Figures and Tables

**Table 1:** Hydrogen-bond geometry (Å, °) *Cg*1 and *Cg*2 are the centroids of the C1–C6 and C8–C13 rings, respectively.

*D*—H⋯*A*	*D*—H	H⋯*A*	*D*⋯*A*	*D*—H⋯*A*
C9—H9⋯*Cg*2^i^	0.93	2.70	3.533 (3)	150
C15—H15*B*⋯*Cg*1^ii^	0.96	2.76	3.581 (4)	142

Symmetry codes: (i) 
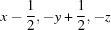
; (ii) 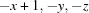.

**Fig. 1.**
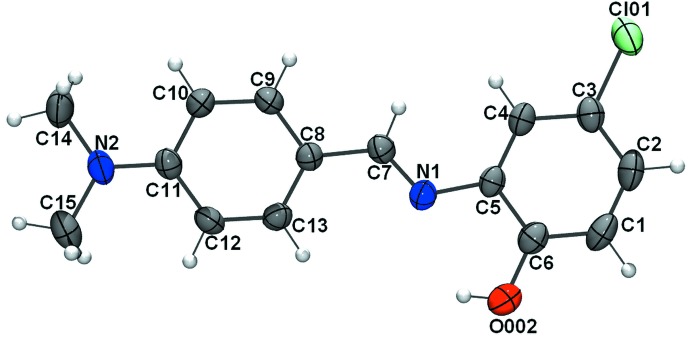
View of the molecular structure of the title compound, with atom labelling. Displacement ellipsoids are drawn at the 50% probability level.

## supplementary crystallographic information

### 1. Comment

Schiff bases are widely used for synthetic purposes both by organic and inorganic chemists (Arora *et al.*, 2002) and have uses as biological, analytical, polymer and liquid crystalline materials (Tanaka & Shiraishi, 2000). Schiff bases are reported to show a variety of biological activities such as antibacterial (Jarrahpour & Khalili, 2006; Jarrahpour *et al.*, 2004; El-masry *et al.*, 2000), antifungal (More *et al.*, 2001; Singh & Dash, 1988), anticancer (Desai *et al.*, 2001; Phatak *et al.*, 2000) and herbicidal activities (Samadhiya & Halve, 2001). As an extension of our work on Schiff bases, we report here the crystal structure of the title compound (I).

### 2. Experimental

A mixture of 3,4-dimethoxyaniline (1 mmol) and 4-nitrobenzaldehyde (1 mmol) was added and heated to form a clear solution. To this a few drops of conc. H2SO4 was added as a catalyst and refluxed for 6 h. After cooling the solution, After stirring at 80°C for 20 min the formed precipitate was filtered off and washed with ice ethanol to give pure Schiff base as an yellow solid in an 80% yield. The crude product was dissolved in ethanol and two spoons of activated charcoal were added. The mixture was filtered over celite&reg; and the product was crystallized from ethyl acetate, yellow crystal was obtained after two weeks.

### 3. Refinement

Anisotropic thermal parameters were applied to all non hydrogen atoms. The organic hydrogen atoms attached to C atoms and N atom were fixed geometrically and treated as riding with C—H = 0.93 Å (aromatic) or 0.96 Å (methyl) and N—H = 0.86 Å with U_iso_(H) = 1.5U_eq_(C) for methyl H atoms and 1.2U_eq_(C,N) for the others.

### Crystal data

**Table:**

C_15_H_15_ClN_2_O	*Z* = 8
*M**_r_* = 274.74	*F*(000) = 1152
Orthorhombic, *P**b**c**a*	*D*_x_ = 1.347 Mg m^−^^3^
Hall symbol: -P 2ac 2ab	Mo *K*α radiation, λ = 0.71073 Å
*a* = 7.411 (5) Å	µ = 0.28 mm^−^^1^
*b* = 12.314 (5) Å	*T* = 293 K
*c* = 29.684 (5) Å	Block, yellow
*V* = 2709 (2) Å^3^	0.03 × 0.02 × 0.01 mm

### Data collection

**Table:**

Bruker APEXII CCD diffractometer	1895 reflections with *I* > 2σ(*I*)
Radiation source: sealed tube	*R*_int_ = 0.028
Graphite monochromator	θ_max_ = 25.1°, θ_min_ = 3.1°
phi and ω scans	*h* = −8→8
14319 measured reflections	*k* = −13→14
2346 independent reflections	*l* = −33→35

### Refinement

**Table:**

Refinement on *F*^2^	Primary atom site location: structure-invariant direct methods
Least-squares matrix: full	Secondary atom site location: difference Fourier map
*R*[*F*^2^ > 2σ(*F*^2^)] = 0.043	Hydrogen site location: inferred from neighbouring sites
*wR*(*F*^2^) = 0.102	H-atom parameters constrained
*S* = 1.10	*w* = 1/[σ^2^(*F*_o_^2^) + (0.0321*P*)^2^ + 1.9647*P*] where *P* = (*F*_o_^2^ + 2*F*_c_^2^)/3
2346 reflections	(Δ/σ)_max_ = 0.001
172 parameters	Δρ_max_ = 0.20 e Å^−^^3^
0 restraints	Δρ_min_ = −0.20 e Å^−^^3^

### Special details

**Table:**

Geometry. Bond distances, angles *etc*. have been calculated using the rounded fractional coordinates. All su's are estimated from the variances of the (full) variance-covariance matrix. The cell e.s.d.'s are taken into account in the estimation of distances, angles and torsion angles
Refinement. Refinement on *F*^2^ for ALL reflections except those flagged by the user for potential systematic errors. Weighted *R*-factors *wR* and all goodnesses of fit *S* are based on *F*^2^, conventional *R*-factors *R* are based on *F*, with *F* set to zero for negative *F*^2^. The observed criterion of *F*^2^ > σ(*F*^2^) is used only for calculating -*R*-factor-obs *etc*. and is not relevant to the choice of reflections for refinement. *R*-factors based on *F*^2^ are statistically about twice as large as those based on *F*, and *R*-factors based on ALL data will be even larger.

### Fractional atomic coordinates and isotropic or equivalent isotropic displacement parameters (Å^2^)

**Table:**

	*x*	*y*	*z*	*U*_iso_*/*U*_eq_	
Cl01	0.50194 (10)	0.14231 (6)	0.23931 (2)	0.0581 (2)	
O002	0.7854 (3)	−0.17647 (13)	0.11151 (6)	0.0568 (6)	
N1	0.6988 (2)	0.01773 (14)	0.07922 (6)	0.0368 (6)	
N2	0.7096 (3)	0.22034 (16)	−0.12244 (6)	0.0488 (7)	
C1	0.7045 (4)	−0.1285 (2)	0.18620 (8)	0.0527 (9)	
C2	0.6370 (4)	−0.0540 (2)	0.21635 (8)	0.0508 (9)	
C3	0.5870 (3)	0.04721 (19)	0.20103 (7)	0.0401 (7)	
C4	0.6031 (3)	0.07596 (18)	0.15641 (7)	0.0377 (7)	
C5	0.6693 (3)	0.00046 (17)	0.12564 (7)	0.0336 (6)	
C6	0.7202 (3)	−0.10202 (19)	0.14121 (7)	0.0415 (8)	
C7	0.6153 (3)	0.09407 (18)	0.05884 (7)	0.0353 (7)	
C8	0.6406 (3)	0.12185 (17)	0.01192 (7)	0.0326 (7)	
C9	0.5587 (3)	0.21453 (18)	−0.00490 (7)	0.0404 (7)	
C10	0.5801 (3)	0.24837 (19)	−0.04866 (7)	0.0409 (7)	
C11	0.6858 (3)	0.18775 (17)	−0.07873 (7)	0.0351 (7)	
C12	0.7676 (3)	0.09286 (18)	−0.06181 (7)	0.0384 (7)	
C13	0.7462 (3)	0.06157 (17)	−0.01784 (7)	0.0364 (7)	
C14	0.6269 (4)	0.3194 (2)	−0.13863 (8)	0.0584 (10)	
C15	0.7819 (4)	0.1475 (2)	−0.15601 (7)	0.0542 (9)	
H1	0.73970	−0.19690	0.19620	0.0630*	
H02	0.78730	−0.15000	0.08620	0.0850*	
H2	0.62520	−0.07170	0.24670	0.0610*	
H4	0.57000	0.14510	0.14690	0.0450*	
H7	0.53250	0.13460	0.07530	0.0420*	
H9	0.48630	0.25550	0.01420	0.0480*	
H10	0.52420	0.31180	−0.05840	0.0490*	
H12	0.83780	0.05050	−0.08090	0.0460*	
H13	0.80310	−0.00110	−0.00770	0.0440*	
H14A	0.65710	0.32970	−0.16980	0.0870*	
H14B	0.67050	0.37980	−0.12130	0.0870*	
H14C	0.49830	0.31420	−0.13550	0.0870*	
H15A	0.78840	0.18430	−0.18450	0.0810*	
H15B	0.70480	0.08520	−0.15870	0.0810*	
H15C	0.90060	0.12460	−0.14720	0.0810*	

### Atomic displacement parameters (Å^2^)

**Table:**

	*U*^11^	*U*^22^	*U*^33^	*U*^12^	*U*^13^	*U*^23^
Cl01	0.0648 (4)	0.0729 (5)	0.0366 (3)	0.0025 (4)	0.0062 (3)	−0.0031 (3)
O002	0.0736 (13)	0.0446 (10)	0.0522 (10)	0.0160 (9)	−0.0035 (9)	0.0047 (8)
N1	0.0422 (11)	0.0344 (10)	0.0337 (9)	−0.0028 (9)	−0.0004 (8)	0.0040 (8)
N2	0.0689 (15)	0.0490 (12)	0.0285 (9)	−0.0023 (11)	0.0040 (10)	−0.0006 (9)
C1	0.0608 (17)	0.0469 (14)	0.0504 (14)	0.0015 (13)	−0.0106 (13)	0.0188 (12)
C2	0.0555 (16)	0.0617 (16)	0.0351 (12)	−0.0045 (14)	−0.0066 (11)	0.0156 (12)
C3	0.0366 (12)	0.0506 (14)	0.0330 (11)	−0.0062 (11)	−0.0021 (10)	0.0026 (10)
C4	0.0390 (13)	0.0384 (12)	0.0357 (11)	−0.0042 (10)	−0.0017 (10)	0.0077 (10)
C5	0.0324 (11)	0.0345 (11)	0.0340 (11)	−0.0054 (10)	−0.0033 (9)	0.0059 (9)
C6	0.0397 (13)	0.0415 (13)	0.0432 (13)	0.0000 (11)	−0.0065 (11)	0.0053 (11)
C7	0.0362 (12)	0.0368 (12)	0.0328 (11)	−0.0023 (10)	0.0025 (10)	−0.0003 (9)
C8	0.0335 (12)	0.0328 (11)	0.0315 (11)	−0.0023 (10)	0.0001 (9)	0.0001 (9)
C9	0.0445 (14)	0.0431 (13)	0.0335 (11)	0.0100 (11)	0.0058 (10)	−0.0018 (10)
C10	0.0485 (14)	0.0392 (13)	0.0350 (11)	0.0100 (11)	−0.0009 (10)	0.0035 (10)
C11	0.0392 (13)	0.0359 (12)	0.0303 (11)	−0.0084 (10)	−0.0005 (9)	−0.0024 (9)
C12	0.0437 (13)	0.0360 (12)	0.0356 (11)	−0.0011 (11)	0.0060 (10)	−0.0084 (10)
C13	0.0408 (13)	0.0287 (11)	0.0398 (12)	−0.0003 (10)	−0.0008 (10)	0.0006 (9)
C14	0.084 (2)	0.0552 (16)	0.0359 (13)	−0.0045 (15)	−0.0009 (13)	0.0107 (11)
C15	0.0613 (17)	0.0677 (17)	0.0335 (12)	−0.0105 (14)	0.0077 (12)	−0.0066 (12)

### Geometric parameters (Å, º)

**Table:**

Cl01—C3	1.749 (3)	C10—C11	1.403 (3)
O002—C6	1.361 (3)	C11—C12	1.409 (3)
O002—H02	0.8200	C12—C13	1.370 (3)
N1—C7	1.278 (3)	C1—H1	0.9300
N1—C5	1.411 (3)	C2—H2	0.9300
N2—C11	1.370 (3)	C4—H4	0.9300
N2—C15	1.444 (3)	C7—H7	0.9300
N2—C14	1.447 (3)	C9—H9	0.9300
C1—C6	1.380 (3)	C10—H10	0.9300
C1—C2	1.376 (4)	C12—H12	0.9300
C2—C3	1.378 (4)	C13—H13	0.9300
C3—C4	1.376 (3)	C14—H14A	0.9600
C4—C5	1.393 (3)	C14—H14B	0.9600
C5—C6	1.396 (3)	C14—H14C	0.9600
C7—C8	1.446 (3)	C15—H15A	0.9600
C8—C13	1.394 (3)	C15—H15B	0.9600
C8—C9	1.386 (3)	C15—H15C	0.9600
C9—C10	1.373 (3)		
			
Cl01···H15A^i^	3.1200	H4···O002^ii^	2.6600
Cl01···H1^ii^	3.0400	H7···C4	2.5700
Cl01···H14A^iii^	2.9500	H7···H4	2.1500
O002···N1	2.655 (3)	H7···H9	2.3700
O002···C10^iv^	3.406 (4)	H7···O002^ii^	2.9000
O002···C7^v^	3.312 (4)	H9···H7	2.3700
O002···H14C^iv^	2.7900	H9···C8^i^	3.0700
O002···H15C^vi^	2.6400	H9···C11^i^	3.0200
O002···H4^v^	2.6600	H9···C12^i^	2.8500
O002···H7^v^	2.9000	H9···C13^i^	2.8700
N1···O002	2.655 (3)	H10···C14	2.5000
N1···H02	2.1800	H10···H14B	2.3200
N1···H13	2.7000	H10···H14C	2.3000
C7···C13^iv^	3.512 (4)	H12···C15	2.5600
C7···O002^ii^	3.312 (4)	H12···H15B	2.5500
C10···O002^iv^	3.406 (4)	H12···H15C	2.2200
C13···C7^iv^	3.512 (4)	H12···H14B^v^	2.4200
C2···H15B^iv^	3.0800	H13···N1	2.7000
C3···H15B^iv^	2.9900	H14A···H15A	2.0800
C4···H15B^iv^	3.0200	H14A···Cl01^ix^	2.9500
C4···H7	2.5700	H14B···C10	2.7800
C6···H15C^vi^	2.8300	H14B···H10	2.3200
C6···H14C^iv^	3.0800	H14B···H12^ii^	2.4200
C7···H4	2.7100	H14C···C10	2.7700
C8···H9^vii^	3.0700	H14C···H10	2.3000
C10···H14B	2.7800	H14C···O002^iv^	2.7900
C10···H14C	2.7700	H14C···C6^iv^	3.0800
C11···H9^vii^	3.0200	H15A···H14A	2.0800
C12···H15B	2.9200	H15A···H2^x^	2.5500
C12···H9^vii^	2.8500	H15A···Cl01^vii^	3.1200
C12···H15C	2.7500	H15B···C12	2.9200
C13···H9^vii^	2.8700	H15B···H12	2.5500
C14···H10	2.5000	H15B···C2^iv^	3.0800
C15···H12	2.5600	H15B···C3^iv^	2.9900
H1···Cl01^v^	3.0400	H15B···C4^iv^	3.0200
H02···N1	2.1800	H15C···C12	2.7500
H2···H15A^viii^	2.5500	H15C···H12	2.2200
H4···C7	2.7100	H15C···O002^vi^	2.6400
H4···H7	2.1500	H15C···C6^vi^	2.8300
			
C6—O002—H02	109.00	C2—C1—H1	120.00
C5—N1—C7	119.88 (18)	C6—C1—H1	120.00
C11—N2—C14	120.47 (19)	C1—C2—H2	120.00
C14—N2—C15	116.85 (18)	C3—C2—H2	120.00
C11—N2—C15	121.31 (19)	C3—C4—H4	120.00
C2—C1—C6	120.2 (2)	C5—C4—H4	120.00
C1—C2—C3	119.1 (2)	N1—C7—H7	118.00
Cl01—C3—C4	118.96 (18)	C8—C7—H7	118.00
C2—C3—C4	121.8 (2)	C8—C9—H9	119.00
Cl01—C3—C2	119.23 (17)	C10—C9—H9	119.00
C3—C4—C5	119.3 (2)	C9—C10—H10	120.00
N1—C5—C4	126.47 (19)	C11—C10—H10	120.00
N1—C5—C6	114.69 (18)	C11—C12—H12	119.00
C4—C5—C6	118.79 (19)	C13—C12—H12	119.00
O002—C6—C5	119.38 (19)	C8—C13—H13	119.00
C1—C6—C5	120.8 (2)	C12—C13—H13	119.00
O002—C6—C1	119.9 (2)	N2—C14—H14A	109.00
N1—C7—C8	124.6 (2)	N2—C14—H14B	109.00
C7—C8—C13	123.85 (19)	N2—C14—H14C	109.00
C9—C8—C13	117.13 (19)	H14A—C14—H14B	110.00
C7—C8—C9	119.01 (19)	H14A—C14—H14C	109.00
C8—C9—C10	122.7 (2)	H14B—C14—H14C	110.00
C9—C10—C11	120.3 (2)	N2—C15—H15A	109.00
N2—C11—C10	121.3 (2)	N2—C15—H15B	109.00
N2—C11—C12	121.7 (2)	N2—C15—H15C	110.00
C10—C11—C12	117.05 (19)	H15A—C15—H15B	109.00
C11—C12—C13	121.5 (2)	H15A—C15—H15C	109.00
C8—C13—C12	121.3 (2)	H15B—C15—H15C	109.00
			
C7—N1—C5—C4	−21.8 (3)	N1—C5—C6—O002	−2.4 (3)
C7—N1—C5—C6	161.0 (2)	N1—C5—C6—C1	177.8 (2)
C5—N1—C7—C8	177.4 (2)	C4—C5—C6—O002	−179.9 (2)
C14—N2—C11—C10	0.6 (3)	C4—C5—C6—C1	0.3 (3)
C14—N2—C11—C12	−178.7 (2)	N1—C7—C8—C9	−172.2 (2)
C15—N2—C11—C10	−165.6 (2)	N1—C7—C8—C13	6.7 (4)
C15—N2—C11—C12	15.1 (3)	C7—C8—C9—C10	178.1 (2)
C6—C1—C2—C3	−0.5 (4)	C13—C8—C9—C10	−0.9 (3)
C2—C1—C6—O002	−179.4 (2)	C7—C8—C13—C12	−178.9 (2)
C2—C1—C6—C5	0.4 (4)	C9—C8—C13—C12	−0.1 (3)
C1—C2—C3—Cl01	−179.8 (2)	C8—C9—C10—C11	1.1 (3)
C1—C2—C3—C4	−0.2 (4)	C9—C10—C11—N2	−179.7 (2)
Cl01—C3—C4—C5	−179.48 (17)	C9—C10—C11—C12	−0.4 (3)
C2—C3—C4—C5	0.9 (4)	N2—C11—C12—C13	178.8 (2)
C3—C4—C5—N1	−178.1 (2)	C10—C11—C12—C13	−0.5 (3)
C3—C4—C5—C6	−1.0 (3)	C11—C12—C13—C8	0.8 (3)

Symmetry codes: (i) *x*−1/2, −*y*+1/2, −*z*; (ii) −*x*+3/2, *y*+1/2, *z*; (iii) *x*, −*y*+1/2, *z*+1/2; (iv) −*x*+1, −*y*, −*z*; (v) −*x*+3/2, *y*−1/2, *z*; (vi) −*x*+2, −*y*, −*z*; (vii) *x*+1/2, −*y*+1/2, −*z*; (viii) −*x*+3/2, −*y*, *z*+1/2; (ix) *x*, −*y*+1/2, *z*−1/2; (x) −*x*+3/2, −*y*, *z*−1/2.

### Hydrogen-bond geometry (Å, º)

Cg1 and Cg2 are the centroids of the C1–C6 and C8–C13 rings, respectively.

**Table:**

*D*—H···*A*	*D*—H	H···*A*	*D*···*A*	*D*—H···*A*
C9—H9···*Cg*2^i^	0.93	2.70	3.533 (3)	150
C15—H15*B*···*Cg*1^iv^	0.96	2.76	3.581 (4)	142

Symmetry codes: (i) *x*−1/2, −*y*+1/2, −*z*; (iv) −*x*+1, −*y*, −*z*.
